# Development and validation of statistical shape models of the primary functional bone segments of the foot

**DOI:** 10.7717/peerj.8397

**Published:** 2020-02-04

**Authors:** Tamara M. Grant, Laura E. Diamond, Claudio Pizzolato, Bryce A. Killen, Daniel Devaprakash, Luke Kelly, Jayishni N. Maharaj, David J. Saxby

**Affiliations:** 1 School of Allied Health Sciences, Griffith University, Gold Coast, QLD, Australia; 2 Griffith Centre for Biomedical and Rehabilitation Engineering, Menzies Health Institute Queensland, Griffith University, Gold Coast, QLD, Australia; 3 Human Movement Biomechanics Research Group, Katholieke Universiteit Leuven, Leuven, Belgium; 4 School of Human Movement and Nutritional Sciences, University of Queensland, Brisbane, QLD, Australia

**Keywords:** Musculoskeletal modelling, Statistical shape modelling, Subject-specific modelling, Foot bones

## Abstract

**Introduction:**

Musculoskeletal models are important tools for studying movement patterns, tissue loading, and neuromechanics. Personalising bone anatomy within models improves analysis accuracy. Few studies have focused on personalising foot bone anatomy, potentially incorrectly estimating the foot’s contribution to locomotion. Statistical shape models have been created for a subset of foot-ankle bones, but have not been validated. This study aimed to develop and validate statistical shape models of the functional segments in the foot: first metatarsal, midfoot (second-to-fifth metatarsals, cuneiforms, cuboid, and navicular), calcaneus, and talus; then, to assess reconstruction accuracy of these shape models using sparse anatomical data.

**Methods:**

Magnetic resonance images of 24 individuals feet (age = 28 ± 6 years, 52% female, height = 1.73 ± 0.8 m, mass = 66.6 ± 13.8 kg) were manually segmented to generate three-dimensional point clouds. Point clouds were registered and analysed using principal component analysis. For each bone segment, a statistical shape model and principal components were created, describing population shape variation. Statistical shape models were validated by assessing reconstruction accuracy in a leave-one-out cross validation. Statistical shape models were created by excluding a participant’s bone segment and used to reconstruct that same excluded bone using full segmentations and sparse anatomical data (i.e. three discrete points on each segment), for all combinations in the dataset. Tali were not reconstructed using sparse anatomical data due to a lack of externally accessible landmarks. Reconstruction accuracy was assessed using Jaccard index, root mean square error (mm), and Hausdorff distance (mm).

**Results:**

Reconstructions generated using full segmentations had mean Jaccard indices between 0.77 ± 0.04 and 0.89 ± 0.02, mean root mean square errors between 0.88 ± 0.19 and 1.17 ± 0.18 mm, and mean Hausdorff distances between 2.99 ± 0.98 mm and 6.63 ± 3.68 mm. Reconstructions generated using sparse anatomical data had mean Jaccard indices between 0.67 ± 0.06 and 0.83 ± 0.05, mean root mean square error between 1.21 ± 0.54 mm and 1.66 ± 0.41 mm, and mean Hausdorff distances between 3.21 ± 0.94 mm and 7.19 ± 3.54 mm. Jaccard index was higher (*P* < 0.01) and root mean square error was lower (*P* < 0.01) in reconstructions from full segmentations compared to sparse anatomical data. Hausdorff distance was lower (*P* < 0.01) for midfoot and calcaneus reconstructions using full segmentations compared to sparse anatomical data.

**Conclusion:**

For the first time, statistical shape models of the primary functional segments of the foot were developed and validated. Foot segments can be reconstructed with minimal error using full segmentations and sparse anatomical landmarks. In future, larger training datasets could increase statistical shape model robustness, extending use to paediatric or pathological populations.

## Introduction

Musculoskeletal models are tools to aid investigation of movement patterns, tissue loading, and neuromechanics (Bahl et al., 2019; Rajagopal et al., 2016; Sommer, Miller & Pijanowski, 1982). Bone anatomy plays an important role in muscle-tendon and articular force estimates through its influence on muscle attachment sites (Suwarganda et al., 2019) and articular geometry (Gerus et al., 2013). The foot is typically modelled as either a single rigid body or multiple rigid segments that represent the rear-, mid-, and fore-foot (Bruening, Cooney & Buczek, 2012; Leardini et al., 2007; Malaquias et al., 2017). Multi-segment foot modelling has improved our understanding of foot function, including foot contribution to power generation and absorption during locomotion (Bruening, Cooney & Buczek, 2012; Kelly, Lichtwark & Cresswell, 2015; Zelik & Honert, 2018), which is not well represented by single rigid body models (Zelik & Honert, 2018). However, studies commonly do not personalise bone geometry (Delp et al., 1990; Prinold et al., 2016), potentially effecting biomechanical analyses (Prinold et al., 2016).

Linear scaling of generic bone templates and creating models from medical imaging are two methods used to personalise bone geometry for musculoskeletal models. Linear scaling orthonormally scales a bone template to match an individual’s anthropometry. Generic bone templates are typically created from one individual or are averaged from a limited dataset (Sommer, Miller & Pijanowski, 1982). Generic models have been found to poorly characterise an individual’s bone geometry of the pelvis and femur. Indeed, reconstruction error for linearly scaled generic models has been found to be more than double that of personalised bone geometries (~14 mm compared to ~6 mm for the femur) (Suwarganda et al., 2019). This lack of fidelity to the individual being studied can potentially reduce the accuracy of subsequent biomechanical analyses, including muscle moment arms and joint contact forces (Bahl et al., 2019; Correa et al., 2011; Gerus et al., 2013; Lerner et al., 2015; Zelik & Honert, 2018; Zhang et al., 2016). Accurate bone geometry can be generated by segmenting medical images (Modenese et al., 2018). However, due to acquisition cost and processing time related to medical imaging (Correa et al., 2011), these methods are impractical for large sample sizes.

Statistical shape modelling is an alternative to medical imaging segmentation that can be used to reconstruct personalised bone geometries (Barratt et al., 2008; Suwarganda et al., 2019). Statistical shape modelling is a mathematical process that determines the shape variation in a training dataset using a statistical procedure (e.g. principal component analysis) that is computationally efficient and robust to artefacts (Zheng, Li & Szekely, 2017). Once established, a statistical shape model can be morphed to fit a known shape (e.g. a bone) (Barratt et al., 2008). Statistical shape models are well-established tools for image analysis (Cootes et al., 1995) and have previously been used to characterise and reconstruct the pelvis, femur, tibia, and patella (Bahl et al., 2019; Baka et al., 2012; Bryan, Nair & Taylor, 2009; Rao et al., 2013; Suwarganda et al., 2019; Zhang & Besier, 2017). On the other hand, few studies have applied statistical shape modelling to the foot bones (Melinska et al., 2017; Melinska et al., 2015). Two previous studies reported a statistical shape model of the cuboid, navicular, calcaneus, and talus, but did not assess model performance (i.e. reconstruction accuracy) (Melinska et al., 2017; Melinska et al., 2015). Statistical shape models of the primary functional segments of the foot have the potential to inform clinical decisions (by well monitoring growth & development and degeneration), prosthesis design, and preoperative planning (Melinska et al., 2017; Melinska et al., 2015). However, before statistical shape models of the foot segments can be used in musculoskeletal models, their ability to accurately reconstruct personalised bone geometries must be established.

The primary aim of this study was to develop and validate statistical shape models of the first metatarsal, midfoot (second-to-fifth metatarsals, medial, intermediate and lateral cuneiforms, cuboid, and navicular), calcaneus, and talus. The secondary aim of this study was to assess the performance of these statistical shape models when supplied with sparse anatomical data, commonly acquired in clinical gait laboratories, to evaluate potential for future clinical use. We used segmented bone geometries from magnetic resonance imaging (MRI) as our gold standard, and compared reconstruction accuracy of statistical shape models using both full segmentations from MRI and sparse anatomical data from commonly used anatomical landmarks (Leardini et al., 2007; Leardini et al., 1999). We hypothesised that the statistical shape models would accurately reconstruct (i.e. high shape similarity compared to gold standard) foot bone geometries using both full segmentations and sparse anatomical data, but reconstructions from full segmentations would be more accurate than those from sparse anatomical data.

## Materials and Methods

### Participants

Twenty-four participants (age = 28.2 ± 5.8 years, 46% female, height = 1.73 ± 0.08 m, mass = 67.1 ± 13.4 kg) underwent bilateral lower limb MRI at one of two imaging clinics (QSCAN, Parkwood Village, QLD, Australia, and Universal Medical Imaging, Calvary Hospital, Bruce, ACT, Australia). Scans at both locations were undertaken with a 3 T Philips Ingenia scanner (Philips Medical Systems, Best, Netherlands). Axial T_1_-weighted three-dimensional fast field echo sequences were acquired from the superior iliac crests to below the toes with participants extended and supine on the imaging gantry. Images were acquired in five stations, consisting of ~252 slices per station with 10 mm overlap between stations. Slice thickness and interslice gap were both 1 mm, and pixel resolution was 0.59 mm × 0.59 mm × 0.59 mm. Participants were enrolled in one of two studies with institutional ethical approval (Griffith Human Research Ethics Committee references 2017/521 and 2017/020). All participants provided written informed consent prior to participating.

### Magnetic resonance image processing

**Figure 1:**
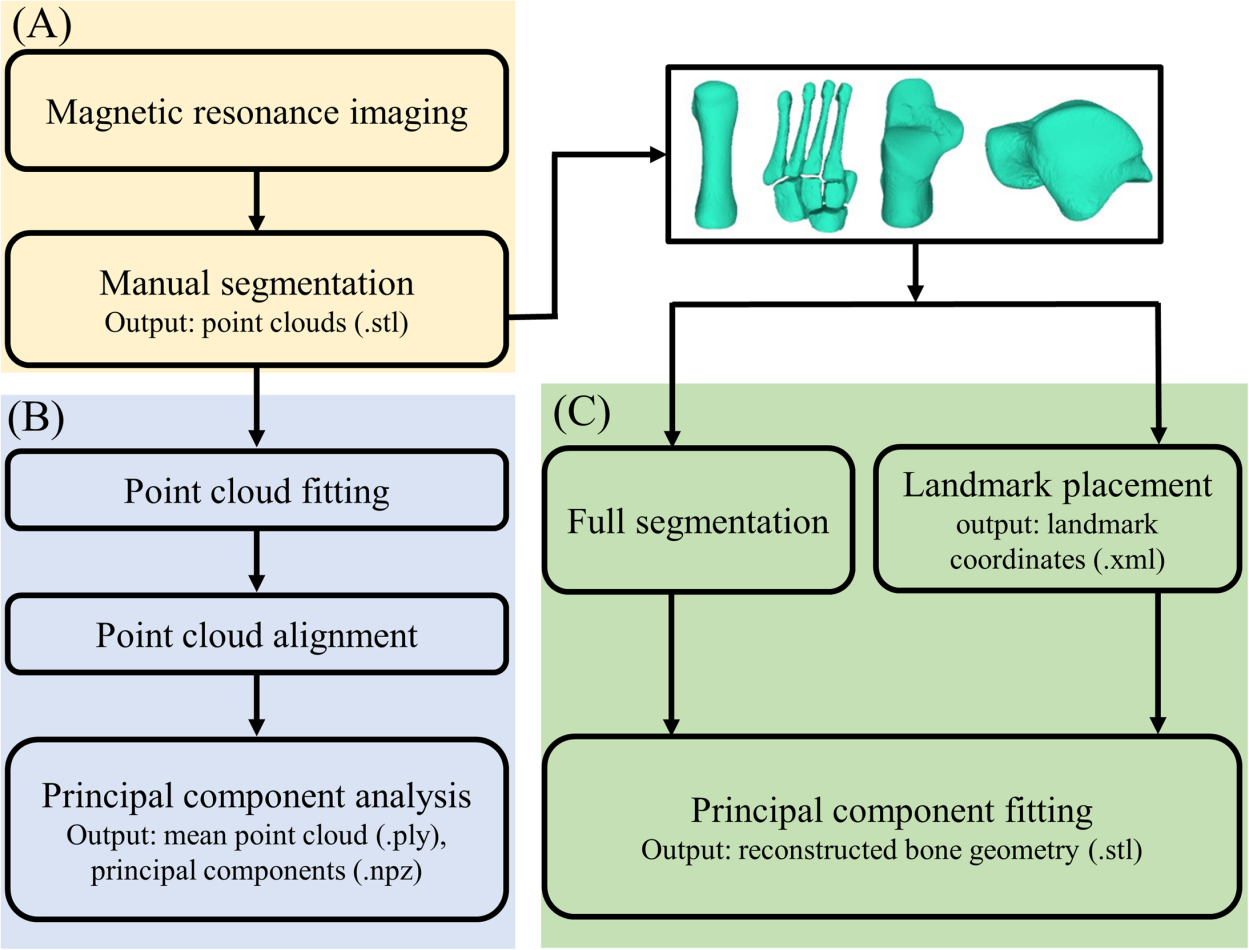
Outline of the process of creating and validating a statistical shape model. (A) Magnetic resonance image processing; (B) statistical shape model generation; and (C) statistical shape model validation.

Magnetic resonance imaging data were processed in two steps (Fig. 1): (1) medical image segmentation, and (2) statistical shape model generation. First, MRI data were segmented in Mimics v20 (Materialise, Leuven, Belgium) to create three-dimensional reconstructions of the four foot bone segments: first metatarsal, midfoot (second-to-fifth metatarsals, medial, intermediate and lateral cuneiforms, cuboid, and navicular), calcaneus, and talus. The talus and calcaneus were considered independently, because the subtalar joint and mid-tarsal joint represent two primary foot articulations. Segmentations were created by manually identifying outer contours of bone segments of interest on each image slice using greyscale thresholding to create masks (Bryan, Nair & Taylor, 2009). The second-to-fifth metatarsals, medial, intermediate and lateral cuneiforms, cuboid, and navicular were segmented as individual bones, but within the same mask (i.e. as bone body: the midfoot). The first metatarsal, calcaneus, and talus were segmented into individual masks. Masks were then wrapped and smoothed following the manufacturer’s guidelines to reduce stair-step artefacts (DeVries et al., 2008), and point clouds exported to stereolithography (STL) format.

### Statistical shape model generation

Second, three-dimensional point clouds were used to create statistical shape models. Point clouds of the left foot bones were mirrored using the mirror function in 3-Matic (Materialise, Leuven, Belgium). Volumetric shape similarity was compared for left and mirrored right point clouds. For each point cloud, a three-dimensional coordinate system was defined consistent with recommendations from the International Society of Biomechanics for ankle joint coordinate systems (Wu et al., 2002). The coordinate system was used to align segmentations, where it was applied to each segmentation digitally using 3-Matic (Materialise, Leuven, Belgium), and extracted as three-dimensional spatial coordinates. A transformation matrix containing a rotation matrix, matrix padding, and three-dimensional spatial coordinates of the native and new positions of the point cloud was created. Point clouds were then transformed about the transformation matrix (Craig, 2005). The GIAS2 toolbox (https://pypi.org/project/gias2/) was then used to register the point clouds from each participant for a specific bone segment (e.g. calcaneus) in two ways. First, a non-rigid registration was performed using radial basis functions. Second, a rigid iterative closest-point registration (Besl & McKay, 1992) was performed. Once registered, a principal component analysis was performed. The principal components represent the variation across the data. A mean point cloud was then created using the first three principal components from the principal component analysis. The mean point cloud and associated principal components could then be used to reconstruct personalised bone geometries.

### Statistical shape model validation

Each of the four statistical shape models (i.e. first metatarsal, midfoot, calcaneus, and talus) were validated by testing reconstruction accuracy in a leave-one-out cross validation. The GIAS2 toolbox was used to reconstruct bone geometries. Mean point clouds were fit to target data (i.e. full segmentations or sparse anatomical landmarks) using rigid body translation, rotation, and deformation along a specified number of principal components (seven or eight for full segmentation reconstructions, or three for the sparse anatomical landmarks), minimising the distance between the target data and mean point cloud. The objective of the optimisation was to minimise distance between target data (either the full segmentations or sparse anatomical landmarks) and the deformed shape model (Zhang, Hislop-Jambrich & Besier, 2016). A penalty weight (i.e. Mahalanobis distance) was used to constrain statistical shape model deformation by quantifying the similarity of the deformed and mean statistical shape models (Suwarganda et al., 2019; Zhang et al., 2016). Mahalanobis distance ranges from 0 to 1, where a value closer to one result in less similarity between the deformed and mean statistical shape models. Mahalanobis distance was set to 0.1, consistent with previous studies (Suwarganda et al., 2019), and was implemented to avoid spurious shape model deformation.

**Figure 2:**
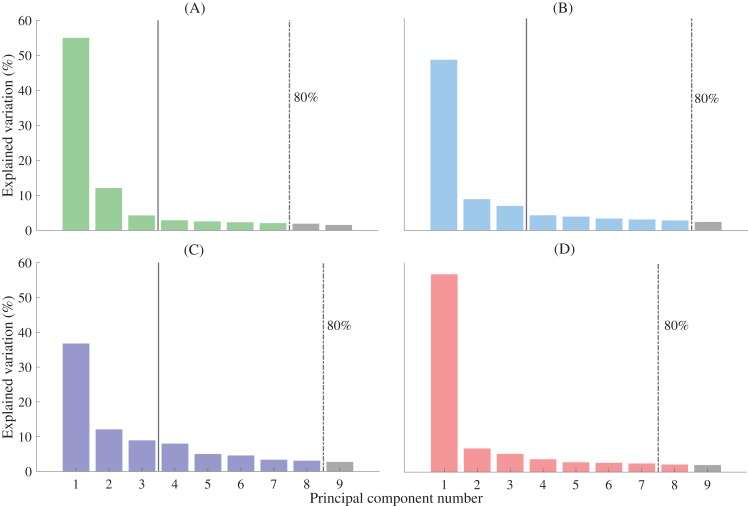
Percentage of shape variation explained by the first nine principal components for each functional segment. (A) First metatarsal; (B) calcaneus; (C) midfoot; and (D) talus. Individual bars represent the percentage of shape variation explained by each principal component. A threshold of 80% total shape variation explained was used to determine the number of principal components used for reconstructions from complete segmentation (dashed vertical line). Three principal components were used for reconstructions generated from sparse anatomical landmarks (solid vertical line). The midfoot segment includes the second-to-fifth metatarsal, cuneiforms (medial, intermediate, and lateral), cuboid, and navicular.

**Figure 3:**
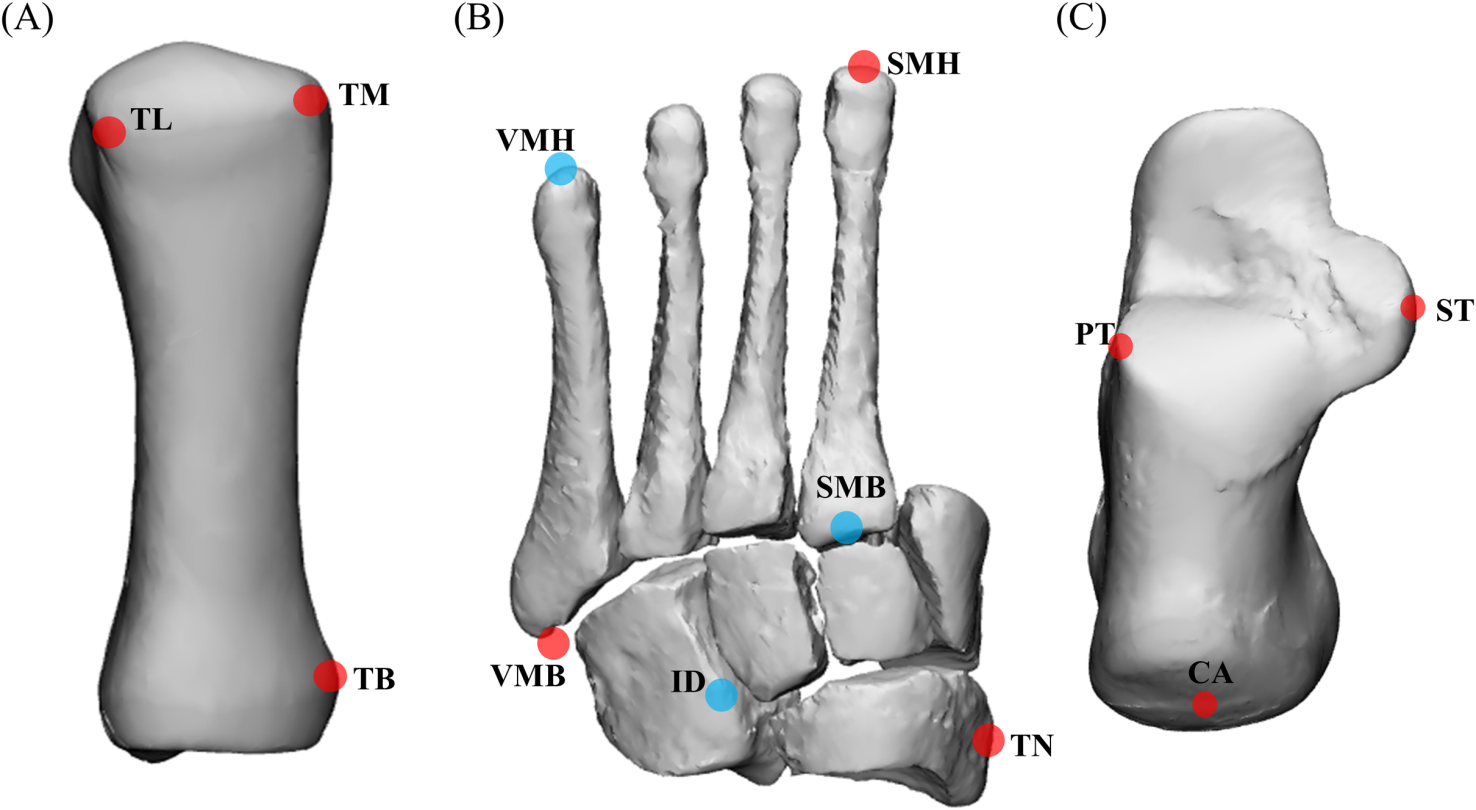
Anatomical landmarks used for sparse reconstructions. (A) First metatarsal; (B) midfoot; and (C) calcaneus. TL = most lateral projection of the head of the first metatarsal; TM = most medial projection of the head of the first metatarsal; TB = most medial projection of the base of the first metatarsal; VMH = head of the fifth metatarsal; SMH = head of the second metatarsal; VMB = base of the fifth metatarsal; SMB = base of the second metatarsal; ID = mid-point between the apex of the tuberosity of the navicular and the base of the fifth metatarsal; TN = most medial apex of the tuberosity of the navicular; PT = lateral apex of the peroneal tubercle of the calcaneus; ST = most medial apex of the sustenaculum tali; and CA = upper central ridge of the posterior surface of the calcaneus. The midfoot segment includes the second-to-fifth metatarsal, cuneiforms (medial, intermediate, and lateral), cuboid, and navicular. Note: (A) based on Leardini et al. (1999), (B) and (C) based on Leardini et al. (2007).

A leave-one-out cross validation was performed to assess reconstruction accuracy. In this validation, a statistical shape model is created by excluding one participant’s bone geometry, then used to reconstruct the bone excluded geometry, and then reconstruction accuracy assessed against excluded geometry. This process is repeated for all possible combinations in the entire dataset (29 first metatarsals, 33 mid-feet, 26 calcanei, 33 tali). Previous studies have used varying numbers of principal components in reconstructions, accounting for a range of population shape variation (Zhang et al., 2016; Zhang et al., 2014). One study selected a threshold of 75% population variation to select principal components for reconstructions (Zhang et al., 2016). To be conservative, the minimum number of principal components that accounted for 80% of shape variance was used for reconstructions from full segmentations in this study (Fig. 2). The performance of the statistical shape model was assessed using the accuracy of reconstructions generated from the X, Y, and Z coordinates of point clouds of full bone segmentations and the X, Y, and Z coordinates of sparse anatomical landmarks. Sparse anatomical landmarks were located on the full segmentations and their coordinates extracted to an Extensible Markup Language (XML) file. The sparse anatomical landmarks included in our method corresponded to the motion capture marker sets commonly used in gait analysis (Fig. 3) (Leardini et al., 2007; Leardini et al., 1999). Three landmarks were selected per bone segment (Leardini et al., 2007; Leardini et al., 1999). Each landmark was identified from manual segmentations for each individual and digitally placed using 3-matic (Materialise, Leuven, Belgium). The talus was excluded from the sparse analysis as it has no externally accessible anatomical landmarks. The sparse anatomical data reconstructions were performed along the first three principal components (Fig. 2). The first three principal components were used because they represented the maximum number that can be used to create unique weightings, given that we have included only three unique anatomical landmarks for each functional segment in the model (Wold, Esbensen & Geladi, 1987).

### Statistical analysis

Volumetric shape similarity between left and mirrored right foot bones for each participant was assessed using Jaccard index (0–1). Accuracy of the reconstructed bone segments was assessed using a nearest neighbour algorithm to calculate Jaccard index, root mean square error (RMSE; mm), and Hausdorff distance (mm). Jaccard index assesses volumetric similarity by quantifying the intersection of two registered volumes (i.e. manual segmentation and statistical shape model reconstruction) relative to the union of two volumes (Taha & Hanbury, 2015), where an index of 1 indicates 100% overlapping volume. The RMSE is a measure of mean error between the reconstructed data (i.e. from full segmentations or sparse anatomical data) and the gold standard (i.e. full segmentations). Hausdorff distance is the maximum distance between two similar points on two registered point clouds (i.e. segmentation and reconstruction) through many-to-many correspondence (Taha & Hanbury, 2015). Shapiro–Wilk tests were used to assess data normality. Jaccard index, RMSE, and Hausdorff distance did not meet the assumptions of normality for any bone segments. Consequently, Wilcoxon Signed-Ranks tests were used to compare accuracy from reconstructions generated from full segmentations and sparse anatomical landmarks. Statistical Package for Social Sciences version 25 (SPSS Inc., Chicago, IL, USA) was used for all statistical analyses. Significance was set at 0.05.

## Results

**Figure 4:**
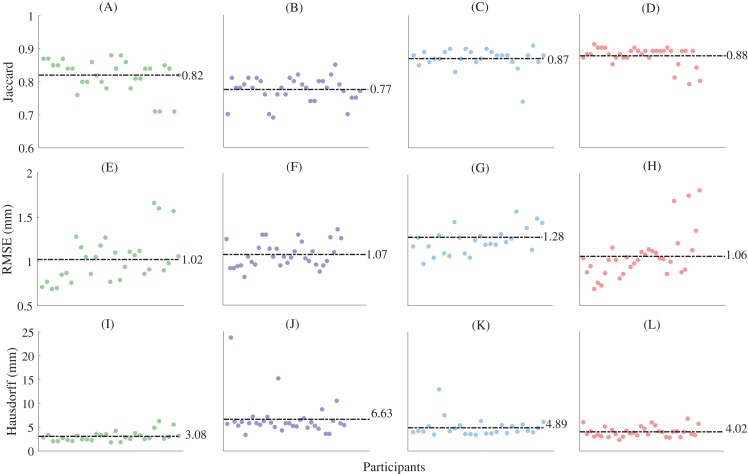
Shape similarity for reconstructions generated from complete magnetic resonance imaging segmentations. (A) First metatarsal Jaccard indices; (B) midfoot Jaccard indices; (C) calcaneus Jaccard indices; (D) talus Jaccard indices; (E) first metatarsal root mean square error; (F) midfoot root mean square error; (G) calcaneus metatarsal root mean square error; (H) talus metatarsal root mean square error; (I) first metatarsal Hausdorff distances; (J) midfoot Hausdorff distances; (K) calcaneus Hausdorff distances; and (L) talus Hausdorff distances. Horizontal dashed lines represent mean values. The midfoot segment includes the second-to-fifth metatarsals, cuneiforms (medial, intermediate, and lateral), cuboid, and navicular. RMSE—root mean square error.

The fitting errors (RMSE) for the non-rigid registration (i.e. the first registration step) were 0.494 ± 0.05 mm, 0.642 ± 0.08 mm, 0.643 ± 1.17 mm, and 0.55 ± 0.12 mm for the first metatarsal, midfoot, calcaneus, and talus, respectively. The fitting errors approach the dimensions of the MRI resolution, and thus can be considered acceptable. The mean Jaccard indices between the left and mirrored right point clouds foot segment point clouds were 0.49 ± 0.14, 0.48 ± 0.1, 0.68 ± 0.12, and 0.76 ± 0.09 for the first metatarsal, midfoot, calcaneus, and talus bone segments, respectively. Principal component analysis revealed a typical decaying slope from low to high numbers of principal components (Fig. 2). Greater than 80% of sample variance was explained by seven principal components in the first metatarsal and talus, and eight principal components in the midfoot and calcaneus. As such, seven and eight principal components were used to reconstruct relevant bones from full segmentations (Fig. 2). Reconstructions generated using the statistical shape models and full segmentations for the first metatarsal, midfoot, calcaneus, and talus had mean Jaccard indices between 0.77 ± 0.04 (midfoot) and 0.88 ± 0.03 (talus), mean RMSE between 1.02 ± 0.26 mm (first metatarsal) and 1.28 ± 0.25 mm (calcaneus), and Hausdorff distances between 3.08 ± 1.04 mm (first metatarsal) and 6.63 ± 3.68 mm (midfoot) (Fig. 4).

**Figure 5:**
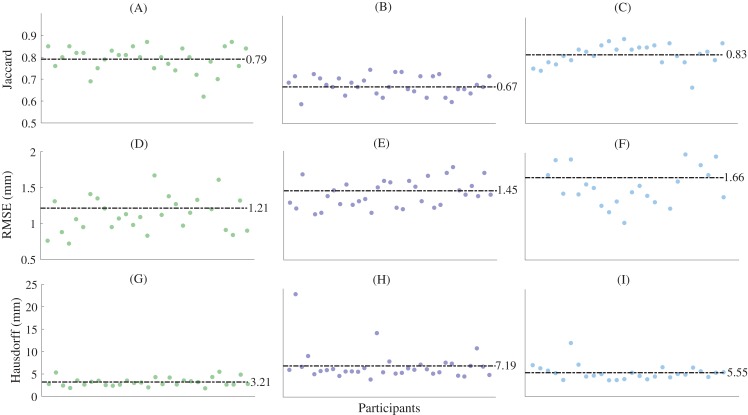
Shape similarity for reconstructions generated from sparse anatomical landmarks. (A) First metatarsal Jaccard indices; (B) midfoot Jaccard indices; (C) calcaneus Jaccard indices; (D) first metatarsal root mean square error; (E) midfoot metatarsal root mean square error; (F) calcaneus root mean square error; (G) first metatarsal Hausdorff distances; (H) midfoot Hausdorff distances; and (I) calcaneus Hausdorff distances. Horizontal dashed lines represent mean values. The midfoot segment includes the second-to-fifth metatarsals, cuneiforms (medial, intermediate, and lateral), cuboid, and navicular. RMSE—root mean square error.

For the reconstructions using sparse anatomical landmarks, the first three principal components from the relevant statistical shape models were used to reconstruct the first metatarsal, midfoot, and calcaneus (Fig. 5). The sum of the shape variation explained from the first three principal components was 71.2%, 57.9%, and 63.8% for the first metatarsal, midfoot, and calcaneus segments, respectively. The reconstructions had mean Jaccard indices between 0.67 ± 0.06 (midfoot) and 0.83 ± 0.05 (calcaneus), mean RMSE between 1.21 ± 0.55 mm (first metatarsal) and 1.66 ± 0.41 mm (calcaneus), and mean Hausdorff distances between 3.21 ± 0.94 mm (first metatarsal) and 7.19 ± 3.54 mm (midfoot) across all bone segments (Table 1).

**Table 1:** Comparison of shape similarity between reconstructions generated from complete magnetic resonance imaging segmentations and sparse anatomical landmarks.

	Jaccard index		*P*-value	RMSE (mm)		*P*-value	Hausdorff distance (mm)		*P*-value
	Complete segmentation	Sparse landmarks		Complete segmentation	Sparse landmarks		Complete segmentation	Sparse landmarks	
First metatarsal	0.82 (0.05)	0.79 (0.06)	<0.01*	1.02 (0.26)	1.21 (0.55)	<0.01*	3.08 (1.04)	3.21 (0.94)	0.55
Midfoot	0.77 (0.04)	0.67 (0.06)	<0.01*	1.07 (0.14)	1.45 (0.30)	<0.01*	6.63 (3.68)	7.19 (3.54)	0.03*
Calcaneus	0.87 (0.03)	0.83 (0.05)	<0.01*	1.28 (0.25)	1.66 (0.41)	<0.01*	4.89 (1.91)	5.55 (1.73)	0.03*

**Notes:**

All values are mean (standard deviation).

RMSE—root mean square error; the midfoot segment includes the second-to-fifth metatarsals, cuneiforms (medial, intermediate, and lateral), cuboid, and navicular.

* Denotes significant differences in shape similarity between reconstructions generated from complete segmentations and sparse anatomical landmarks using a Wilcoxon Signed-Ranks test.

**Figure 6:**
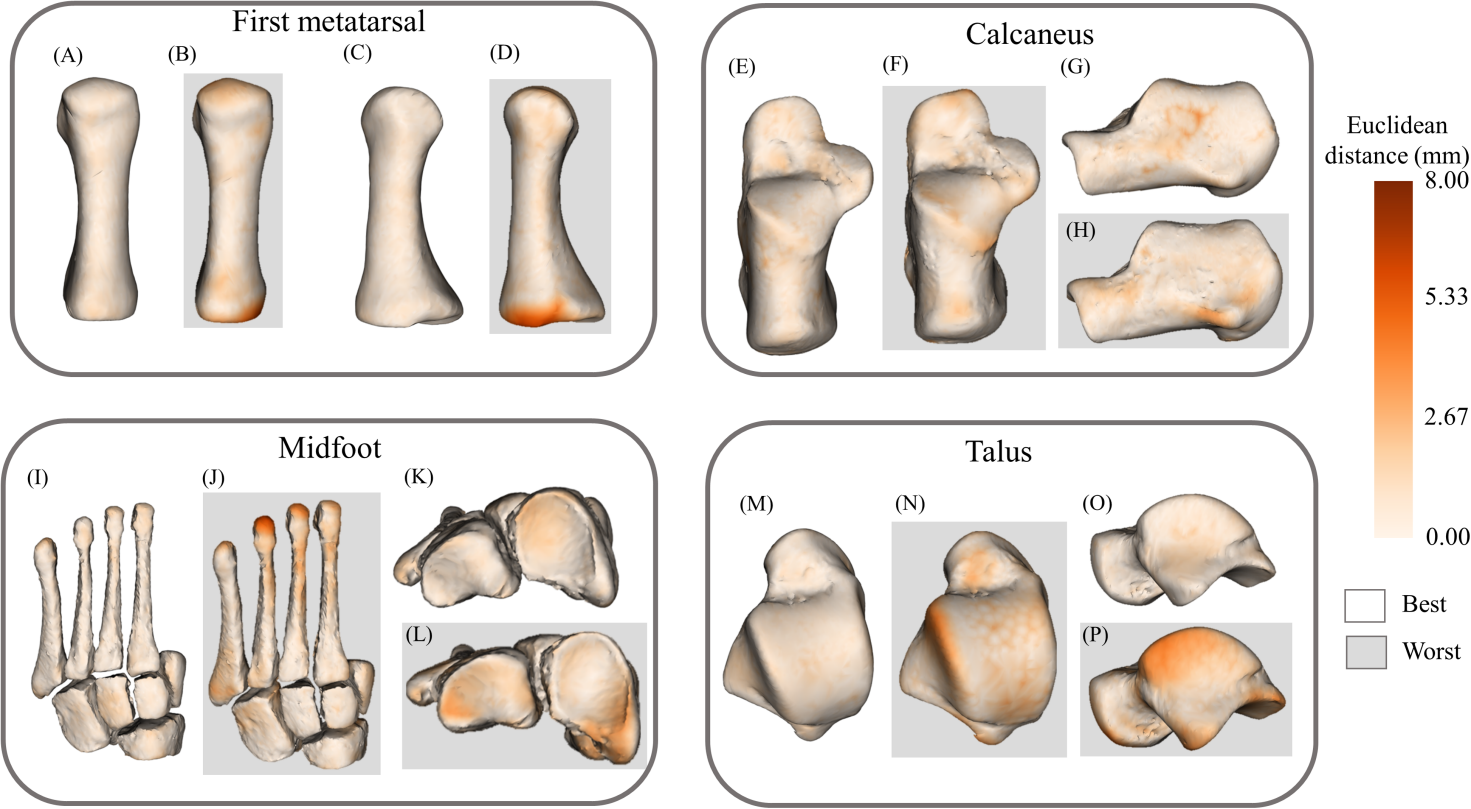
Best- and worst-case reconstructions generated from full magnetic resonance imaging (MRI) segmentations in terms of absolute Euclidean distance (mm). (A) Plantar view of best first metatarsal reconstruction; (B) plantar view of worst first metatarsal reconstruction; (C) medial view of best first metatarsal reconstruction; (D) medial view of worst first metatarsal reconstruction; (E) plantar view of best calcaneus reconstruction; (F) plantar view of worst calcaneus reconstruction; (G) lateral view of best calcaneus reconstruction; (H) lateral view of worst calcaneus reconstruction; (I) plantar view of best midfoot reconstruction; (J) plantar view of worst midfoot reconstruction; (K) proximal view of best midfoot reconstruction; (L) proximal view of worst midfoot reconstruction; (M) plantar view of best talus reconstruction; (N) plantar view of worst talus reconstruction; (O) lateral view of best talus reconstruction; and (P) lateral view of worst talus reconstruction. Non-shaded background: best-case reconstruction from the leave-one-out cross validation; and shaded background: worst-case reconstruction from the leave-one-out cross validation. Euclidean distance is represented by the colour map which ranges from 0 mm (cream) to 8 mm (brown). The midfoot segment includes the second-to-fifth metatarsals, cuneiforms (medial, intermediate, and lateral), cuboid, and navicular bones.

**Figure 7:**
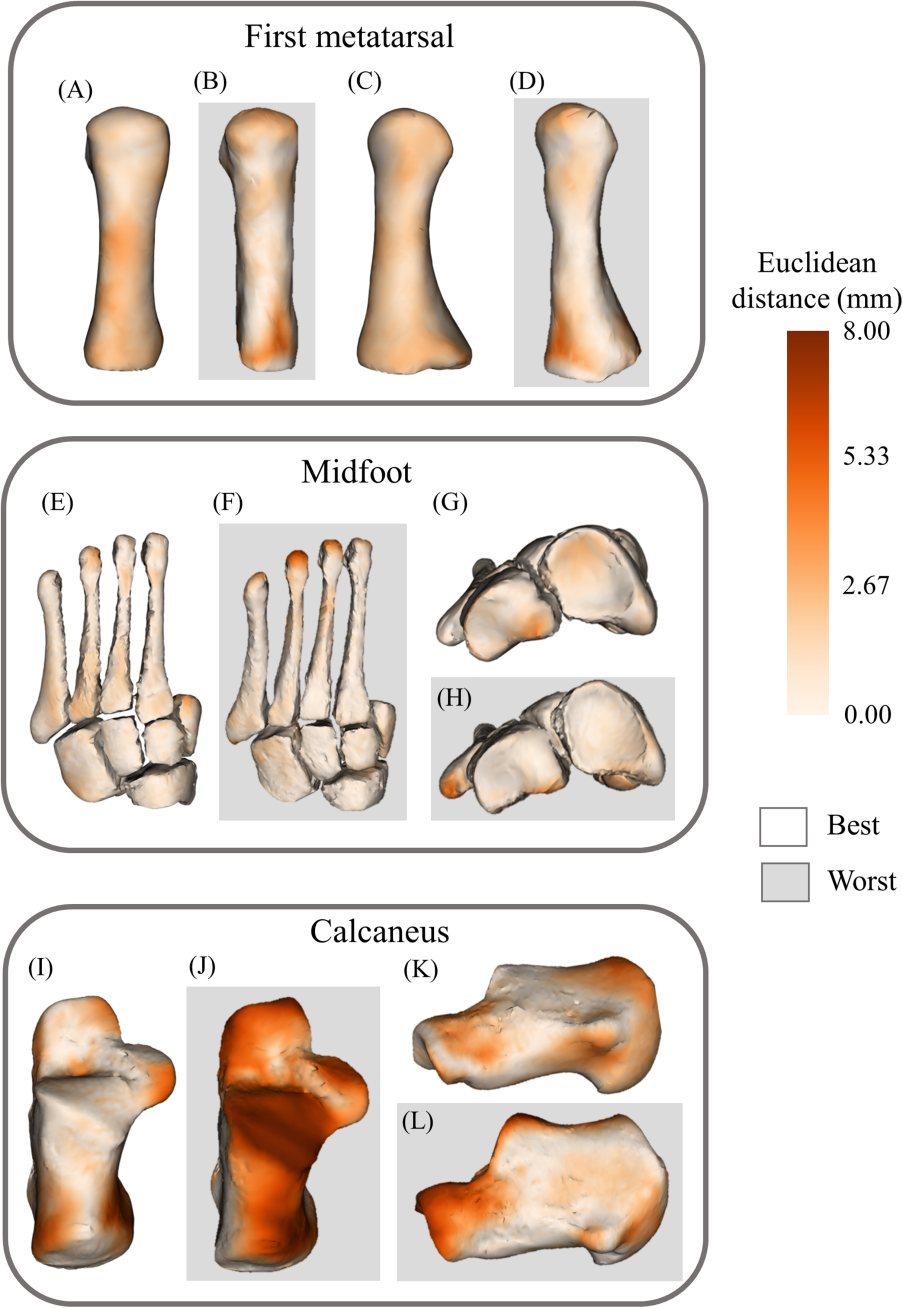
Best- and worst-case reconstructions generated from sparse anatomical data in terms of absolute Euclidean distance (mm). (A) Plantar view of best first metatarsal reconstruction; (B) plantar view of worst first metatarsal reconstruction; (C) medial view of best first metatarsal reconstruction; (D) medial view of worst first metatarsal reconstruction; (E) plantar view of best midfoot reconstruction; (F) plantar view of worst midfoot reconstruction; (G) proximal view of best midfoot reconstruction; (H) proximal view of worst midfoot reconstruction (I) plantar view of best calcaneus reconstruction; (J) plantar view of worst calcaneus reconstruction; (K) lateral view of best calcaneus reconstruction; and (L) lateral view of worst calcaneus reconstruction. Non-shaded background: best-case reconstruction from the leave-one-out cross validation; and shaded background: worst-case reconstruction from the leave-one-out cross validation. Euclidean distance is represented by the colour map which ranges from 0 mm (cream) to 8 mm (brown). The midfoot segment includes the second-to-fifth metatarsals, cuneiforms (medial, intermediate, and lateral), cuboid, and navicular bones.

The statistical shape models using full segmentations generated significantly more accurate bone reconstructions (Fig. 4) for the first metatarsal, midfoot, and calcaneus compared to those generated from sparse anatomical landmarks in terms of Jaccard indices (*P* < 0.01) and RMSE (*P* < 0.01) (Table 1). The statistical shape models using full segmentations generated significantly more accurate bone reconstructions of the calcaneus (*P* = 0.03) and midfoot (*P* = 0.03) in terms of Hausdorff distance. The best- and worst-case reconstruction examples for the reconstructions generated from full segmentation in terms of point-to-point (Euclidean) distance are presented visually in Fig. 6. The best- and worst-case reconstruction examples for the reconstructions generated from sparse anatomical landmarks in terms of Euclidean distance are presented visually in Fig. 7.

## Discussion

This study is the first to develop and validate statistical shape models of the primary functional segments of the foot. For statistical shape models to be useful, they must accurately reconstruct bone geometries without a requirement for time-consuming and costly medical imaging. Our statistical shape models were able to reconstruct bone geometries from sparse anatomical landmarks, albeit with lower accuracy than those generated from full segmentations. Statistical shape models, such as those presented in this study, may be an effective way to personalise representations of the functional segments of the foot in a musculoskeletal model. Future studies may consider comparing personalised bone geometries from these statistical shape models to generic scaled models establishing their suitability for biomechanical analyses.

First metatarsal, calcaneus, and talus geometries generated using statistical shape models and full MRI segmentations had excellent accuracy (>80% volumetric similarity), consistent with previous reports focused on other lower limb bones (Suwarganda et al., 2019). Reconstructions of the midfoot segment had lower volumetric similarity (Jaccard index = 0.77) compared to the other analysed bone segments, which is unsurprising given the midfoot segment is markedly more complex (nine bones) compared to segments consisting of a single bone. Previous studies (Zhang & Besier, 2017) have reported femur reconstructions from statistical shape models with lower error than we found for the primary foot bones. This discrepancy may relate to their comparatively large training datasets (*n* = 204) (Zhang & Besier, 2017) and the lesser geometric complexity of the femur compared to the foot bones. Statistical shape models of the navicular, cuboid, calcaneus, and talus have been created (Melinska et al., 2017; Melinska et al., 2015) using spherical harmonics (a set of orthonormal basis functions that can be used to characterise three-dimensional objects) (Heimann & Meinzer, 2009; Schönefeld, 2005) rather than principal component analysis. These prior foot-focused studies presented results for a subset of the foot-ankle complex (navicular, cuboid, calcaneus, and talus) with the aim of a future finite-element modelling application. These studies did not assess the reconstruction accuracy of their statistical shape model, thus preventing direct comparison with our results. Other approaches have been used to develop statistical shape models of the foot bones (Melinska et al., 2017; Melinska et al., 2015); however, to the authors’ knowledge, this is the first study to validate the reconstruction accuracy of statistical shape models of the primary functional segments of the foot.

Foot bone geometries reconstructed using sparse anatomical landmarks yielded shape similarities consistent with previous studies of lower limb bones, where motion capture landmarks reconstructed femur and pelvis geometries with Jaccard indices of ~0.68 and 0.41, respectively (Suwarganda et al., 2019). Pelvis and femur bone geometries generated using a statistical shape model and sparse anatomical data acquired from skin-surface markers remain superior to linearly-scaled generic models in terms of RMSE and Jaccard index (Suwarganda et al., 2019), in addition to accuracy of muscle moment arms and joint contact force estimates (Correa et al., 2011; Gerus et al., 2013; Lerner et al., 2015).

Previous studies have used incomplete segmentations of medical imaging to reconstruct lower limb bone geometries (Suwarganda et al., 2019) and sparse anatomical landmarks to estimate lower limb position and muscle attachment sites (Zhang et al., 2016). Our statistical shape models were able to reconstruct the first metatarsal, midfoot, and calcaneus bone segments using sparse anatomical landmarks, producing Jaccard indices between 0.67 and 0.83. Previous statistical shape models of the foot (Melinska et al., 2017; Melinska et al., 2015) have not been used in conjunction with sparse anatomical landmarks, thus this paper presents the first foray into this area. The lower reconstruction accuracy observed when using sparse anatomical landmarks compared to full segmentation may relate to less information provided to the model for reconstruction (fewer number of principal components used—three vs. seven or eight) and could potentially be improved if additional information was provided to the model, for example additional anatomical landmarks or anthropometric measurements. We excluded the talus from our sparse landmark analysis as it had no externally accessible anatomical landmarks. In future, scaling a statistical shape model of the talus using anthropometric data (e.g. foot length) may be investigated as an alternative to using costly medical imaging such as MRI. Alternatively, three-dimensional ultrasound may provide a fast, affordable, and accessible alternative to acquire critical sparse data of an individual’s musculoskeletal anatomy, including data that is otherwise inaccessible (e.g. regarding the talus) without expensive MRI or ionising radiation from X-ray (Cong et al., 2017; Raum et al., 2014).

Statistical shape models can be used to characterise natural shape variation in anatomy not well represented by commonly used generic bone templates (Bryan, Nair & Taylor, 2009; Zhang & Besier, 2017), but without the cost, time, and potential risk associated with medical imaging. The statistical shape models presented in this study can be used in conjunction with sparse anatomical landmarks to reconstruct personalised models of the primary functional segments of the foot with minimal error. Using sparse anatomical landmarks to create personalised geometric bone models can improve biomechanical analyses by increasing model precision of the individual being studied without requirement for laborious manual image segmentation (Krähenbühl et al., 2017; Suwarganda et al., 2019). Statistical shape models of the primary functional segments of the foot also have a range of applications in the medical field, including improving preoperative planning and postoperative assessment, monitoring growth in children and degeneration of bone in individuals with conditions such as osteoarthritis, and assessing implant placement (Melinska et al., 2015).

This study has limitations that should be considered. First, the phalanges were not included, although the relevance of personalising these structures for subsequent biomechanical analysis of locomotion is unclear. Second, the talus segment was not reconstructed using sparse anatomical landmarks due to a lack of externally accessible landmarks. As mentioned above, future investigations should develop a method to reconstruct talus geometry using less costly medical imaging (e.g. three-dimensional ultrasound) (Devaprakash et al., 2019; Treece et al., 2003). Ultrasound can also provide a much higher image resolution than routine MRI scans (Kositsky et al., 2020). Third, in a preliminary analysis (data not reported) we observed RMSE similar to previous reports when using the number of principal components corresponding to 75% of sample variance, thus we did not explore any effects of varying the number of principal components (Zhang et al., 2016). Fourth, anatomical landmarks used in sparse reconstructions were digitally placed directly to the bone surface of three-dimensional reconstructions. In future, if skin-surface markers are used (e.g. from a clinical gait laboratory), protocols to limit soft tissue sliding and ensure quality anatomical landmark identification should be developed, and their effects on bone reconstruction accuracy assessed. Fifth, there are some regional anatomical errors in reconstructions, particularly in those created from sparse anatomical data (e.g. the posterior talar articular surface of the calcaneus, and the dorsal portion of the calcaneus) (Fig. 7). These regional errors are not present in all reconstructions, however they are a consideration for future applications and subsequent analyses. Sixth, the statistical shape models in the present study were created using data from healthy adults. It is unclear whether these shape models can be extended to children or people with bone pathologies that have abnormal bone geometry. Seventh, these statistical shape models were created using the feet of 24 individuals (29 first metatarsals, 33 mid-feet, 26 calcanei, 33 tali). Including more data in the training set will increase the robustness of the models.

## Conclusions

Results show the primary functional segments of the foot can be reconstructed with minimal error using segmentations from MRI. Importantly, a subset of the foot bone segments can be reconstructed with minimal error using sparse anatomical landmarks that are consistent with skin-surface marker locations from clinical motion capture methods. Statistical shape models could reduce the need for expensive medical imaging and subsequent tedious manual image segmentation.

## Supplemental Information

10.7717/peerj.8397/supp-1 Supplemental Information 1 Individual participant information.

## Additional Information and Declarations

### Data Availability

The following information was supplied regarding data availability: The data is available at Zenodo: Grant, Tamara; Diamond, Laura; Pizzolato, Claudio; Killen, Bryce; Devaprakash, Daniel; Kelly, Luke; Maharaj, Jayishni; Saxby, David. (2019). Development and validation of statistical shape models of the primary functional bone segments of the foot. (Data set). Zenodo. DOI 10.5281/zenodo.3464747.
