# Supplementary material for: Development and validation of statistical shape models of the primary functional bone segments of the foot
Source: PeerJ. 2020 Feb 4;8:e8397. doi: 10.7717/peerj.8397 (PMC7006516; doi:10.7717/peerj.8397)
Supplement: Supplemental Information 1 [file peerj-08-8397-s001.docx]

| Subject number | Sex | Age (years) | Height (cm) | Weight (kg) |
| --- | --- | --- | --- | --- |
| A01 | M | 41 | 187 | 75.8 |
| A09 | M | 19 | 174.7 | 61.0 |
| A10 | F | 27 | 164 | 67.0 |
| A11 | F | 27 | 164 | 54.0 |
| A12 | F | 27 | 163.8 | 52.2 |
| A14 | M | 39 | 184 | 78.5 |
| A17 | M | 28 | 178.5 | 69.7 |
| A18 | M | 23 | 168 | 57.0 |
| A19 | M | 24 | 178 | 69.2 |
| A20 | F | 19 | 172.5 | 57.3 |
| M01 | M | 24 | 182 | 82.0 |
| M02 | F | 22 | 172 | 63.0 |
| M03 | M | 23 | 180 | 88.0 |
| M04 | M | 29 | 178 | 73.45 |
| M05 | F | 34 | 177 | 64.0 |
| M06 | M | 31 | 184 | 100.0 |
| M07 | M | 32 | 185 | 89.0 |
| M08 | M | 34 | 174 | 78.0 |
| M09 | M | 31 | 161 | 45.0 |
| M10 | F | 31 | 163 | 58.0 |
| M11 | F | 21 | 160.5 | 55.0 |
| M12 | F | 26 | 164.5 | 52.0 |
| M13 | F | 29 | 176.2 | 65.0 |
| M14 | F | 36 | 167.1 | 56.0 |
|  |  |  |  |  |
|  | Mean | 28.20833 | 173.2833 | 67.08958 |
|  | STD Dev | 5.794963 | 8.166633 | 13.42473 |

**Supplementary table 1.** Individual participant information.
